# Nanosized-Selenium-Application-Mediated Cadmium Toxicity in Aromatic Rice at Different Stages

**DOI:** 10.3390/plants13162253

**Published:** 2024-08-14

**Authors:** Baoling Cui, Haowen Luo, Xiangbin Yao, Pipeng Xing, Sicheng Deng, Qianqian Zhang, Wentao Yi, Qichang Gu, Ligong Peng, Xianghai Yu, Changjian Zuo, Jingjing Wang, Yangbo Wang, Xiangru Tang

**Affiliations:** 1 State Key Laboratory for Conservation and Utilization of Subtropical Agro-Bioresources, College of Agriculture, South China Agricultural University, Guangzhou 510642, China; 2 Scientific Observing and Experimental Station of Crop Cultivation in South China, Ministry of Agriculture and Rural Affairs, Guangzhou 510642, China; 3 Guangzhou Key Laboratory for Science and Technology of Fragrant Rice, Guangzhou 510642, China; 4 Green Huinong Biotechnology (Shenzhen) Co., Ltd., Shenzhen 518107, China; 5 Shenzhen Agricultural Science and Technology Promotion Center, Shenzhen 518000, China

**Keywords:** cadmium, nano-selenium, aromatic rice, 2-acetyl-1-pyrroline, yield

## Abstract

Cadmium (Cd) pollution restricts the rice growth and poses a threat to human health. Nanosized selenium (NanoSe) is a new nano material. However, the effects of NanoSe application on aromatic rice performances under Cd pollution have not been reported. In this study, a pot experiment was conducted with two aromatic rice varieties and a soil Cd concentration of 30 mg/kg. Five NanoSe treatments were applied at distinct growth stages: (T1) at the initial panicle stage, (T2) at the heading stage, (T3) at the grain-filling stage, (T1+2) at both the panicle initial and heading stages, and (T1+3) at both the panicle initial and grain-filling stages. A control group (CK) was maintained without any application of Se. The results showed that, compared with CK, the T1+2 and T1+3 treatments significantly reduced the grain Cd content. All NanoSe treatments increased the grain Se content. The grain number per panicle, 1000-grain weight, and grain yield significantly increased due to NanoSe application under Cd pollution. The highest yield was recorded in T3 and T1+3 treatments. Compared with CK, all NanoSe treatments increased the grain 2-acetyl-1-pyrroline (2-AP) content and impacted the content of pyrroline-5-carboxylic acid and 1-pyrroline which are the precursors in 2-AP biosynthesis. In conclusion, the foliar application of NanoSe significantly reduced the Cd content, increased the Se content, and improved the grain yield and 2-AP content of aromatic rice. The best amendment was applying NanoSe at both the panicle initial and grain-filling stages.

## 1. Introduction

In the context of the current global environmental crisis, the issue of Cd pollution, which is a pervasive heavy metal pollution problem, cannot be overlooked. Its potential threat to human health, ecosystems, and agricultural production demands urgent attention. Cd is a highly bio-accumulative and toxic metal that can be enriched through the food chain, ultimately causing long-term effects on human health, such as kidney damage and bone disease [1,2,3,4]. Rice (*Oryza sativa* L.) is an important food crop grown globally, which can absorb Cd from the soil through the root system [5,6]. Considering rice is a principal component of the Chinese diet [7], the issue of its Cd contamination is of paramount importance and urgency [8]. Selenium (Se) is an essential trace element that participates in the catalytic reactions of a variety of biological enzymes and possesses significant antioxidant function [9], and this antioxidant function could effectively resist the damage caused by external environmental stresses on living organisms [10,11]. Previous studies showed the efficacy of Se in mitigating the contamination of rice by Cd [12,13,14,15]. In recent years, nanosized Se (NanoSe) has been demonstrated to have a considerable potential in applied research in agriculture, due to its distinctive physicochemical properties, including a high specific surface area, good biocompatibility, and low toxicity [15]. In particular, the efficacy of this approach in mitigating the heavy metal contamination of crops has attracted considerable attention [16,17,18].

Aromatic rice is known for its unique aroma and flavor. However, areas where aromatic rice is cultivated are also threatened by Cd pollution which not only affects the yield and quality of aromatic rice but also endangers human health through the food chain [19]. Therefore, how to effectively mitigate the Cd contamination of aromatic rice and ensure its safe production has become an urgent problem. Previous studies have shown that exogenous substances such as calcium, nitrate, Se, silicon, and melatonin can alleviate the Cd contamination in aromatic rice to different degrees and improve its aroma content and yield [20,21,22,23]. NanoSe is a new form of Se which also is the red elemental Se in powder form. However, the application of NanoSe in aromatic rice production has rarely been reported and the effects of NanoSe application on aromatic rice performances under Cd toxicity are unknown.

Exogenous Se reduces Cd toxicity in the following way: First, it reduces the uptake of Cd from the soil by rice. It has been shown that the application of Se at the tillering stage will produce the maximum number of iron spots at the tasseling stage, which can inhibit the uptake of Cd by the root system [24]. Then, the transfer of Cd to the above-ground parts is reduced. It has also been shown that the foliar application of Se can reduce the transfer of Cd from roots to stems as well as from stems to brown rice [25]. Furthermore, Se application could alleviate Cd stress by improving the antioxidant system including superoxide dismutase (SOD), peroxidase (POD), and catalase (CAT) [15,20,26,27]. The results of Guo et al. [28] also showed that the formation of insoluble Cd–Se complexes reduced the bioavailability of Cd and Se in solution and inhibited the uptake and translocation of Cd and Se in plants.

Therefore, we hypothesized that the foliar application of NanoSe could mediate the negative effects of Cd on aromatic rice plants and conducted a pot experiment with five NanoSe treatments and two aromatic rice cultivars under the conditions of soil Cd content at 30 mg/kg. The objective of this study was to explore the effects of NanoSe application on yield formation, 2-acetyl-1-pyrroline (2-AP, the key component of aromatic rice aroma) biosynthesis, and the Cd uptake of aromatic rice.

## 2. Results

### 2.1. Effect of NanoSe Application on Cd Content of Aromatic Rice

**Figure 1:**
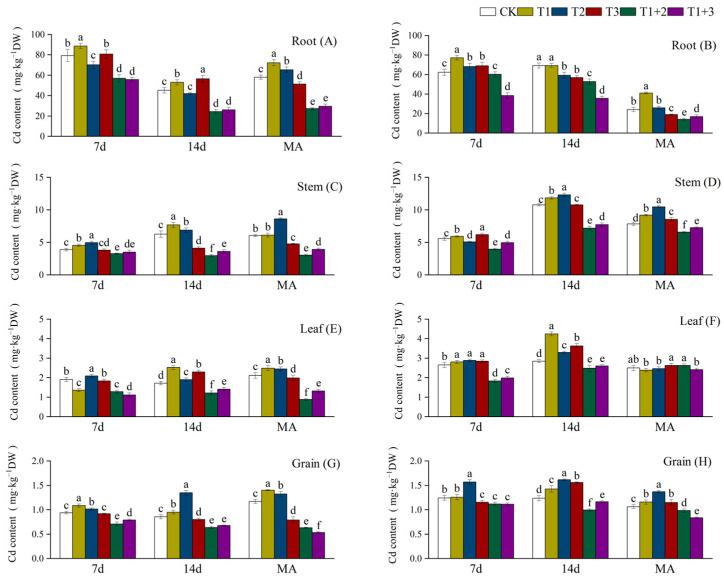
Effect of NanoSe application on Cd content of aromatic rice. The bars represent the means of four repetitions, and the bars sharing a common letter do not differ significantly at *p* ≤ 0.05 according to the LSD test: (**A**) root Cd content of 19X; (**B**) root Cd content of Q19; (**C**) stem Cd content of 19X; (**D**) stem Cd content of Q19; (**E**) leaf Cd content of 19X; (**F**) leaf Cd content of Q19; (**G**) grain Cd content of 19X; and (**H**) grain Cd content of Q19.

The foliar application of NanoSe substantially decreased the Cd content in aromatic rice plants (Figure 1). In roots, the lowest Cd content was observed under T1+2 treatment for 19xiang (19X), which was 40.71% lower than CK. T1+3 treatment Q19 exhibited the lowest Cd content for Qingxiangyou19xiang (Q19), which was 41.45% lower than CK. In stems, leaves, and grains, T1+2 treatment exhibited the lowest Cd content for the both 19X and Q19. The T2 and T3 treatments did not result in a clear reduction in Cd content in the non-edible parts of the rice. From a temporal perspective, the Cd content in the roots decreased gradually as the rice matured, while the Cd content in the stems increased. The Cd content in the leaves and grains did not remarkably change through time. The Cd content was highest in the roots, followed by the stems, leaves, and grains.

### 2.2. Effect of NanoSe Application on Se Content of Aromatic Rice

**Figure 2:**
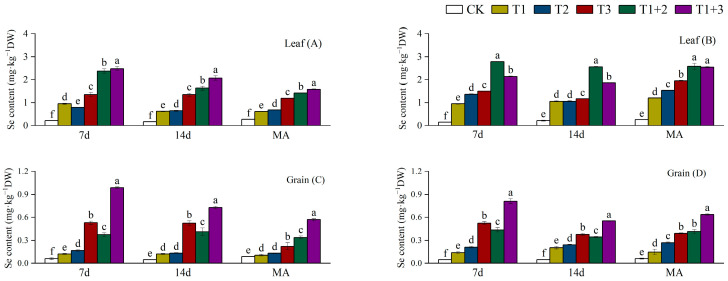
Effect of NanoSe application on Se content of aromatic rice. The bars represent the means of four repetitions, and the bars sharing a common letter do not differ significantly at *p* ≤ 0.05 according to the LSD test: (**A**) leaf Se content of 19X; (**B**) leaf Se content of Q19; (**C**) grain Se content of 19X; and (**D**) grain Se content of Q19.

The foliar application of NanoSe substantially increased the Se content in aromatic rice plants (Figure 2). Relative to CK, all NanoSe treatments significantly increased the leaf Se content, and the highest leaf Se content was recorded in the T1+3 and T1+2 treatments for 19X and Q19, respectively. NanoSe treatments significantly increased the grain Se content by 78.00–1055.00% and the highest grain Se content was recorded in the T1+3 treatment for both varieties.

### 2.3. Effect of NanoSe Application on the Transfer Coefficients of Cd and Se in Aromatic Rice

As illustrated in Table 1, there was no discernible change in the transport coefficients of Cd at each part during the three periods of 19X, whilst, for Q19, the TF root-stem (Cd) exhibited a gradual increase with maturity, while the TF stem-leaf (Cd) and TF stem-grain (Cd) demonstrated a gradual decline with maturity. Compared with CK, the T3 and T1+3 treatments significantly increased the TF leaf-grain (Se) by 57.81% and 51.92%, respectively, for 19X. Moreover, 12.58%- and 19.06%-higher TF leaf-grain (Se) were recorded in the T3 and T1+3 treatments than in CK for Q19.

### 2.4. Effect of NanoSe Application on 2-AP Content of Aromatic Rice

**Figure 3:**
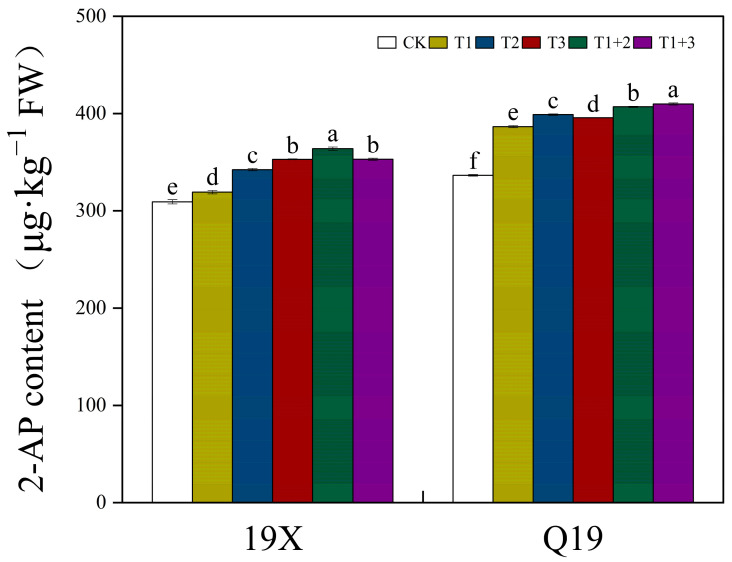
Effect of NanoSe application on 2-AP content of aromatic rice. The bars represent the means of four repetitions, and the bars sharing a common letter do not differ significantly at *p* ≤ 0.05 according to the LSD test.

The foliar application of NanoSe substantially increased the 2-AP content of aromatic rice (Figure 3). For 19X, compared with CK, the T1+2 and T1+3 treatments significantly increased 2-AP content by 17.74% and 14.20%, respectively. For Q19, compared with CK, the T1+2 and T1+3 treatments significantly increased 2-AP content by 20.92% and 21.85%, respectively.

### 2.5. Effect of NanoSe Application on Precursors of 2-AP Biosynthesis of Aromatic Rice

**Figure 4:**
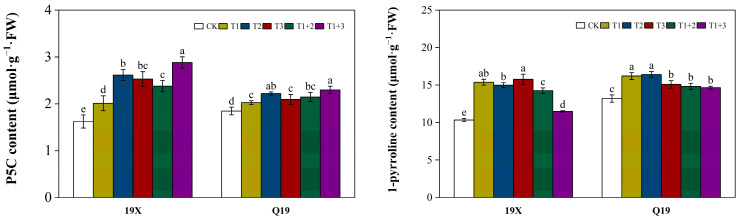
Effect of NanoSe application on content P5C and 1-pyrroline in aromatic rice. The bars represent the means of four repetitions, and the bars sharing a common letter do not differ significantly at *p* ≤ 0.05 according to the LSD test.

As shown in (Figure 4), the foliar application of NanoSe significantly increased the content of P5C and 1-pyrroline for both varieties. The highest P5C content was recorded in the T1+3 treatment for both varieties. The highest or equally highest 1-pyrroline content was recorded in the T1 treatment, followed by the T2 and T3 treatments for both varieties.

### 2.6. Effect of NanoSe Application on Grain Yield and Yield-Related Traits of Aromatic Rice

The foliar application of NanoSe substantially improved the yield formation of aromatic rice (Table 2). Compared with CK, the T1+3 treatment significantly increased the grain number per panicle and 1000-grain weight by 23.34% and 24.61%, respectively, for 19X. For Q19, a 13.23%- and 12.33%-higher grain number per panicle were recorded in the T2 and T3 treatments than in CK. Moreover, a 11.66%-higher 1000-grain weight was also recorded in the T1+2 treatment than in Q19. Moreover, compared with CK, the T2, T3, and T1+3 treatments increased the grain yield by 33.18%, 66.54%, and 66.26%, respectively, for 19X.

## 3. Discussion

### 3.1. Effects of NanoSe on Cd and Se Content

The threat of Cd to human health has been extensively studied [8]. Cd toxicity can severely and negatively affect the photosynthesis and yield of rice plants [29,30,31]. The present study showed the effects of the foliar application of NanoSe on Cd content in aromatic rice plants under Cd pollution and it was found that NanoSe application significantly reduced Cd content in each part of aromatic rice plants. Our results were similar to previous studies that showed that both soil and foliar applications of Se can be effective in mitigating Cd toxicity [32,33]. NanoSe offers greater advantages over common inorganic Se compounds, with a higher biological activity and solubility than inorganic Se [34]. Other benefits of NanoSe over elemental Se forms include reduced toxicity, higher glutathione peroxidase, thioredoxin reductase activity, and excellent catalytic efficiency, and the effect is significantly valued [35,36]. Se alleviates Cd stress mainly by affecting the Cd uptake in roots and transport to the ground [24], and secondarily by alleviating the persecution of the antioxidant system [37]. In this study, the results showed that all NanoSe treatments could effectively reduce the Cd concentration in aromatic rice, while the T1+2 and T1+3 treatments exhibited the best effects on reducing Cd content. On the other hand, the T1, T2, and T3 treatments increased the content of Cd in other non-usable parts higher, possibly due to the lower concentration of Se [38]. It was found that the root uptake of Cd was relatively accumulated during the grain-filling stage and the remobilization of Cd accumulated in rice before the grain-filling stage was very limited [39]. It has also been shown that Cd started to accumulate in grains within 16 days after flowering and the Cd uptake would enhance on 7 days after flowering [40,41]. In the present study, the foliar application of NanoSe at each stage was helpful in inhibiting the Cd uptake of aromatic rice, and the best inhibiting effects were observed when NanoSe was applied twice, i.e., the T1+2 and T1+3 treatments.

Moreover, we observed a higher value of the TF root-stem (Cd) in Q19 than 19X, which indicated that Q19 has a higher transfer capacity of Cd from root to stem. An earlier study showed that low-Cd rice was more tolerant of Cd, and the root-to-aboveground transport of Cd is faster [42]. 19X is the restoration line (parent) of Q19, while a previous study showed that cross-breeding can produce low-Cd rice varieties [33]. Therefore, it can be speculated that Q19 in this study may be a low-Cd-accumulating variety, the specifics of which need to be further explored. The results of this study showed that the Se transfer rates of T3 and T1+3 were significantly higher than other nano-Se treatments, and, thus, we deduced that exogenous NanoSe could be more easily absorbed by aromatic rice when the application occurs after the heading stage. However, the future studies should be carried out at the physiological level to reveal the mechanism of Cd in aromatic rice under the regulation of NanoSe.

### 3.2. Influence of NanoSe on 2-AP Synthesis

2-AP is the source of the distinctive flavor of aromatic rice [43] and there are two pathways for 2-AP biosynthesis, one enzymatic and the other non-enzymatic. During the non-enzymatic pathway, proline, glutamate, and ornithine are converted to 1-pyrroline-5-carboxylic acid (P5C) by Δ1-pyrroline-5-carboxylic acid synthetase (P5CS), proline dehydrogenase (PDH), and ornithine aminotransferase (OAT) enzymes, and Δ1-pyrroline is further converted to 2-AP by a non-enzymatic reaction with methylglyoxal [44,45]. Cd, however, affects its synthesis [46]. Previous studies showed that Na₂SeO₃ application at the heading stage significantly increased the 2-AP content in fragrant rice grains [23,47]. The foliar application of EDTA-Se also significantly increased the grain 2-AP content in fragrant rice, which could be attributed to the improvement in proline (Pro), 1-pyrroline, and corresponding transforming enzymes (e.g., ProDH, DAO, and OAT) [48]. The results of this study showed that the contents of precursors for 2-AP synthesis (P5C and pyrroline) in grains were all significantly increased under NanoSe treatments, suggesting that NanoSe may facilitate the 2-AP biosynthesis in aromatic rice. Furthermore, the 2-AP biosynthesis is very complicated and involves a lot of genes as well as the metabolic pathways. Thus, more studies should be conducted on the molecular level to reveal the regulation mechanism of NanoSe on 2-AP formation in aromatic rice.

### 3.3. Effects of NanoSe on Yield Formation

High concentrations of Cd can impede rice growth, affect its morphological and physiological development, reduce plant biomass, and trigger yield reduction [19]. However, exogenous Se addition has been shown to be effective in mitigating the negative effects of Cd on rice [33]. Se could enhance photosynthesis, specifically by increasing the net photosynthetic rate, intercellular CO₂ concentration, and transpiration efficiency, to improve the grain yield of rice [49]. In particular, Se application at the beginning of the tillering period significantly increased the seed-setting rate, 1000-grain weight, and grain yield of fragrant rice [48]. Our findings showed that the foliar application of NanoSe significantly increased the grain yield of fragrant rice under Cd stress, which could be explained by increased 1000-grain weight and grain number per panicle, which was consistent with a previous study [50]. Moreover, the yield formation depended on the growth and development of rice plants. The limitation of the present study is the lack of monitoring the growth dynamic including photosynthesis, dry matter accumulation, and morphology parameters. Therefore, more studies should be carried out focusing on the growth and development of fragrant rice under the NanoSe application.

## 4. Materials and Methods

### 4.1. Plant Materials and Experimental Design

The pot experiment was conducted in the greenhouse of the experimental farm of the College of Agriculture, South China Agricultural University, Guangzhou, China (23°17′ N, 113°37′ E, 12 m above sea level). This location is situated within the subtropical monsoon humid climate zone, with an average air temperature of 23 °C and an annual precipitation of 2033 mm in 2023. The test soil was a sandy loam with an organic matter content of 11.20 g·kg^−1^, a total nitrogen content of 1.08 g·kg^−1^, an alkaline nitrogen content of 96.61 mg·kg^−1^, a quick-acting phosphorus content of 5.07 mg·kg^−1^, a quick-acting potassium content of 30.99 mg·kg^−1^, and a pH value of 5.22. Two aromatic rice varieties, Qiangxiangyou19xiang (Q19) and 19xiang (19X), were used as the plant materials. The seeds were obtained from the laboratory of the Silk Seedling Rice Science and Innovation Centre of South China Agricultural University. These two rice varieties are well-recognized for their special aroma and are widely planted in South China.

Each pot (21 cm diameter and 23 cm height) contained 10 kg of air-dried, 5 mm-sieved dry soil. Exogenous Cd in the form of CdCl_2_-2.5H_2_O was formulated into a solution of the appropriate concentration and applied into a plastic bucket to control the soil Cd concentration to 30 mg·kg^−1^. A special rice fertilizer, which contained 10% organic fertilizer, 26% urea, 50% superphosphate, 12% potassium chloride, and 1.9% zinc sulfate, was applied at 10.0 g per pot before transplanting. After sowing and nursery raising, the aromatic rice seedlings were transplanted into pots with five plants per pot. Six treatments were adopted as follows:

CK: No NanoSe was applied;
T1: NanoSe was foliar-applied at the panicle initial stage (45 days after the transplanting);
T2: NanoSe was foliar-applied at the heading stage (70 days after the transplanting);
T3: NanoSe was foliar-applied at the grain-filling stage (78 days after the transplanting);
T1+2: NanoSe was foliar-applied at both panicle initial stage and heading stage;
T1+3: NanoSe was foliar-applied at both panicle initial stage and grain-filling stage;
The NanoSe was provided by Green Huinong Biotechnology (Shenzhen) Co., Ltd., Shenzhen, China. The applied Se concentration was 6.67 mg L^−1^ in each treatment.

### 4.2. Sample Collection and Analysis

#### 4.2.1. Determination of Se and Cd Content

The aromatic rice plants from each treatment were collected on 7 and 14 days after the last foliar application and maturity. The plants were divided into four parts: roots, stems, leaves, and spikes. Then, the samples were placed in 105 °C for 30 min, and then at 80 °C to constant weight.

The above samples were ground with a grinder, and then sieved through a 100-mesh sieve to obtain a fine powder for elemental determination. Weigh 0.200 g of the samples into a decoction tube, and 7.5 mL of concentrated hydrochloric acid and 2.5 mL of concentrated nitric acid were added into the tube, and the sample was left to be decocted overnight with a shaking motion. On the next morning, the sample was heated in a 40-hole hot plate digestion furnace (HYP-340, Shanghai Fibre Inspection, Shanghai, China), and the programmer was set at 90 °C for 50 min, 100 °C for 50 min, and 120 °C for 50 min. At the end, the tube was taken out, and cooled down, and then 5 mL of perchloric acid was added. Heating again, the program is 120 °C, 40 min; 190 °C, 200 min; and evaporate until the liquid is transparent and clear or the residue is white to grey-white at the precipitate end. After cooling, the decoction tube was rinsed with ultrapure water several times, and the reinstate was filtered through medium-speed qualitative filter paper, and the filtrate was volume-determined to 25 mL. The liquid to be tested was used to determine the cadmium (228.8 nm) and absorbance value by an atomic absorption spectrophotometer (AA6300C, Shimadzu, Kyoto, Japan), and to make a standard curve for calculating the cadmium content of the sample. Each batch of elimination had a blank control.

#### 4.2.2. Calculation of the Transfer Factor of Se and Cd

The transfer capacity between different parts of rice is expressed by the transfer factors (TFs) which were calculate as follows:

$$\begin{array}{c} \mathrm{TF}\mathrm{root}-\mathrm{stem}\left(\mathrm{Cd}\right)=\frac{\mathrm{Cd}\mathrm{content}\mathrm{in}\mathrm{stem}}{\mathrm{Cd}\mathrm{content}\mathrm{in}\mathrm{root}}\\ \mathrm{TF}\mathrm{stem}-\mathrm{grain}\left(\mathrm{Cd}\right)=\frac{\mathrm{Cd}\mathrm{content}\mathrm{in}\mathrm{grain}}{\mathrm{Cd}\mathrm{content}\mathrm{in}\mathrm{stem}}\\ \mathrm{TF}\mathrm{stem}-\mathrm{leaf}\left(\mathrm{Cd}\right)=\frac{\mathrm{Cd}\mathrm{content}\mathrm{in}\mathrm{leaf}}{\mathrm{Cd}\mathrm{content}\mathrm{in}\mathrm{stem}}\\ \mathrm{TF}\mathrm{leaf}-\mathrm{grain}\left(\mathrm{Se}\right)=\frac{\mathrm{Se}\mathrm{content}\mathrm{in}\mathrm{grain}}{\mathrm{Se}\mathrm{content}\mathrm{in}\mathrm{leaf}} \end{array}$$

#### 4.2.3. Determination of 2-Acetyl-1-Pyrroline (2-AP) and Its Biosynthetic Precursor

At the grain-filling stage, the fresh grains of aromatic rice were collected and used for the determination of pyrroline-5-carboxylic acid (P5C), and 1-pyrroline content. The determination of P5C and 1-pyrroline was carried out according to Zhang et al. [51]. At the maturity stage, the fresh grains of aromatic rice were collected and used for the determination of 2-AP which is the key volatile in aromatic rice aroma. The grain sample was ground into powder at low temperature, we weighed 2 g of the ground sample into a clamp bottle, 10 mL of chromatographically pure dichloromethane was added, and the seal was closed, and the cap was tightened with a slight shock and shaken well. Label the bottle with the treatment number and the repetition number, and put the bottle into an ultrasonic machine at 40 KHZ, 40 °C, and ultrasonic for 240 min. After the sample bottle was cooled down to room temperature and an appropriate amount of anhydrous sodium sulfite was added, the supernatant was immediately aspirated with a 1 mL sterile syringe and injected into the headspace bottle through an organic needle filter membrane (pore size of 0.22 µm, 13 mm), and 0.2 mg kg^−1^ of 2, 4, 6-Trimethylpyridine (TMP) was added as the internal standard, which was then immediately subjected to gas chromatography. The sample was analyzed by GCMS-QP 2010 Plus. The relative amount of aroma 2-AP was calibrated by GCMS-QP 2010 Plus (Shimadzu, Japan) and 2, 4, 6-trimethylpyrimidine (TMP) internal standard method [52].

#### 4.2.4. Determination of Grain Yield

Plants were harvested at maturity to determine the grain yield. The number of effective panicles per plant was calculated and averaged in each treatment. The panicles were collected and manually threshed to calculate and average the grain number per panicle and seed-setting rate. Three thousand filled grains were randomly selected and weighed from each treatment to obtain the 1000-grain weight.

### 4.3. Data Statistics and Analysis

Microsoft Excel 2010 and SPSS 26 software were used for data collection and analysis. Graphs were generated using Origin 2023 software. Multiple comparisons were made using the least significant difference (LSD) test at a significance level of *p* < 0.05.

## 5. Conclusions

The present study firstly investigated the effects of NanoSe application on aromatic rice performances under Cd pollution. The results showed that the foliar application of NanoSe significantly reduced the Cd content in the root, leaf, stem, and grain of aromatic rice under Cd pollution. The Se content also increased due to the NanoSe application. The highest grain Se content and the lowest grain Cd content were recorded in the T1+2 and T1+3 treatments. The highest yield was recorded in the T3 and T1+3 treatments. NanoSe application also increased the content of 2-AP and its precursors including P5C and 1-pyrroline. More studies should be conducted at the molecular level to reveal the alleviation mechanism of NanoSe on Cd toxicity.

## Figures and Tables

**Table 1:** Effect of NanoSe application on Cd and Se transfer coefficients of aromatic rice.

Date	Treatment	19X				Q19			
		TF Root-Stem (Cd)	TF Stem-Leaf (Cd)	TF Stem-Grain (Cd)	TF Leaf-Grain (Se)	TF Root-Stem (Cd)	TF Stem-Leaf (Cd)	TF Stem-Grain (Cd)	TF Leaf-Grain (Se)
7DAL	CK	4.90 ± 0.16 c	49.08 ± 1.58 a	24.14 ± 1.26 a	27.97 ± 6.61 b	8.93 ± 0.05 b	47.68 ± 0.33 b	22.38 ± 1.25 c	32.88 ± 0.99 b
	T1	5.12 ± 0.26 c	29.91 ± 0.69 d	23.93 ± 1.25 a	13.07 ± 0.88 d	7.68 ± 0.07 c	47.32 ± 1.09 b	21.24 ± 0.40 c	14.68 ± 1.55 c
	T2	7.09 ± 0.49 a	41.96 ± 0.30 b	20.44 ± 0.84 c	20.95 ± 1.44 c	7.48 ± 0.39 c	56.68 ± 1.60 a	30.88 ± 0.40 a	15.56 ± 0.89 c
	T3	4.71 ± 0.32 c	48.41 ± 0.74 a	24.20 ± 0.89 a	39.30 ± 1.35 a	8.97 ± 0.60 b	46.01 ± 0.61 b	18.70 ± 0.96 d	35.02 ± 1.51 b
	T4	5.75 ± 0.20 b	38.27 ± 2.26 c	21.66 ± 0.64 bc	15.65 ± 0.95 d	6.61 ± 0.14 d	46.04 ± 2.51 b	28.16 ± 0.27 b	15.65 ± 1.14 c
	T5	6.30 ± 0.48 b	29.96 ± 0.11 d	22.57 ± 1.29 ab	39.71 ± 0.55 a	12.92 ± 0.41 a	40.08 ± 1.65 c	22.41 ± 1.11 c	37.87 ± 1.52 a
14DAL	CK	13.86 ± 0.31 b	27.46 ± 2.42 d	13.66 ± 0.85 c	28.61 ± 1.25 c	15.61 ± 0.59 d	26.40 ± 0.24 c	11.45 ± 0.20 e	21.82 ± 1.25 c
	T1	14.52 ± 0.67 b	32.83 ± 1.88 c	12.37 ± 0.60 c	19.88 ± 0.70 d	17.09 ± 0.35 c	35.87 ± 0.27 a	12.05 ± 0.29 d	19.19 ± 2.21 d
	T2	16.34 ± 0.41 a	27.73 ± 1.87 d	19.62 ± 0.33 b	20.67 ± 0.76 d	20.78 ± 0.80 a	26.78 ± 0.37 c	13.13 ± 0.14 c	22.95 ± 0.14 c
	T3	7.27 ± 0.26 d	55.94 ± 1.07 a	19.49 ± 1.17 b	38.87 ± 2.41 a	18.91 ± 0.46 b	33.70 ± 0.86 b	14.49 ± 0.12 a	32.56 ± 0.96 a
	T4	12.21 ± 0.93 c	41.28 ± 3.41 b	21.62 ± 1.07 a	25.33 ± 3.36 c	13.66 ± 0.67 e	34.50 ± 1.04 ab	13.84 ± 0.65 b	13.41 ± 0.20 e
	T5	14.00 ± 0.59 b	39.03 ± 1.62 b	18.63 ± 1.25 b	35.20 ± 0.82 b	21.75 ± 1.40 a	33.68 ± 1.60 b	15.05 ± 0.22 a	29.77 ± 0.41 b
MA	CK	10.42 ± 0.09 c	35.00 ± 2.37 b	19.30 ± 0.74 c	16.49 ± 1.40 c	32.59 ± 2.61 d	31.78 ± 0.54 bc	13.53 ± 0.07 b	23.10 ± 2.88 ab
	T1	8.45 ± 0.18 e	40.77 ± 1.4 a	22.95 ± 0.67 a	17.43 ± 2.09 c	22.37 ± 0.54 e	25.84 ± 0.57 d	12.56 ± 0.23 c	12.35 ± 3.12 e
	T2	13.16 ± 0.33 a	28.40 ± 0.49 c	15.35 ± 0.64 d	19.23 ± 0.68 c	40.37 ± 2.15 c	23.53 ± 0.84 e	13.08 ± 0.33 bc	17.56 ± 0.58 cd
	T3	9.29 ± 0.27 d	41.73 ± 2.39 a	16.59 ± 0.92 d	37.14 ± 3.86 a	44.92 ± 1.58 ab	30.72 ± 1.45 c	13.45 ± 0.40 b	20.00 ± 0.16 bc
	T4	11.19 ± 0.72 b	28.98 ± 1.09 c	20.81 ± 0.95 b	23.82 ± 1.21 b	46.43 ± 1.79 a	39.86 ± 1.64 a	14.96 ± 0.64 a	16.03 ± 0.72 d
	T5	12.75 ± 0.71 a	33.72 ± 0.57 b	13.54 ± 0.37 e	36.10 ± 1.03 a	42.68 ± 2.58 bc	33.30 ± 0.36 b	11.53 ± 0.30 d	24.98 ± 0.79 a

Note: Values are the means of four repetitions. Values sharing a common letter within a column do not differ significantly at *p* ≤ 0.05 according to the LSD test. “DAL” means days after the last foliar application in the whole experiment.

**Table 2:** Effects of NanoSe on the grain yield of aromatic rice.

Cultivar	Treatment	Effective Panicle Number per Plant	Grains Number per Panicle	Seed-Setting Rate (%)	1000-Grain Weight (g)	Grain Yield (g/pot)
19X	CK	7.33 ± 0.58 a	174.67 ± 6.00 b	61.27 ± 0.02 c	20.04 ± 1.34 c	78.40 ± 4.89 c
	T1	7.00 ± 0.00 a	17506 ± 10.44 b	62.28 ± 0.01 c	22.13 ± 1.26 ab	84.27 ± 1.20 c
	T2	7.67 ± 0.58 a	171.27 ± 5.97 b	67.62 ± 0.02 b	23.55 ± 1.19 a	104.41 ± 8.26 b
	T3	7.33 ± 0.58 a	215.43 ± 7.42 a	75.47 ± 0.04 a	21.32 ± 0.89 bc	126.65 ± 4.85 a
	T1+2	7.67 ± 0.58 a	187.15 ± 6.60 b	55.36 ± 0.02 d	21.47 ± 0.42 bc	85.03 ± 2.46 c
	T1+3	7.67 ± 0.58 a	217.65 ± 3.60 a	66.58 ± 0.01 b	23.55 ± 0.42 a	130.35 ± 6.93 a
Q19	CK	6.33 ± 0.58 c	171.17 ± 5.14 b	55.49 ± 0.06 a	21.62 ± 0.73 c	64.77 ± 5.84 d
	T1	7.67 ± 0.58 b	172.70 ± 5.88 b	57.01 ± 0.08 a	23.79 ± 0.63 a	89.14 ± 8.42 c
	T2	8.00 ± 0.00 ab	193.81 ± 7.83 a	60.39 ± 0.00 a	22.34 ± 0.15 bc	104.58 ± 4.20 ab
	T3	6.33 ± 0.58 c	192.27 ± 8.47 a	59.20 ± 0.06 a	23.27 ± 0.37 ab	105.23 ± 10.41 ab
	T1+2	7.67 ± 0.58 b	156.17 ± 3.34 c	60.31 ± 0.02 a	24.14 ± 0.90 a	94.68 ± 7.59 bc
	T1+3	8.00 ± 0.00 ab	177.39 ± 6.91 b	63.93 ± 0.01 a	22.42 ± 0.98 bc	114.30 ± 3.50 a

Note: Values are the means of four repetitions. Values sharing a common letter within a column do not differ significantly at *p* ≤ 0.05 according to the LSD test.

## Data Availability

Data are contained within the article.
